# A concise synthesis of *anti*-bicyclo[6.1.0]nonyne carboxylic acid†

**DOI:** 10.1039/d4ra06708h

**Published:** 2024-11-21

**Authors:** Mackenzie Weir, Beatrice Vaccari, Aidan Matthews, Graeme Turnbull, Valery N. Kozhevnikov

**Affiliations:** a Department of Applied Sciences, Northumbria University Ellison Building Newcastle upon Tyne NE1 8ST UK valery.kozhevnikov@northumbria.ac.uk; b Dipartimento di Chimica dell'Università degli Studi di Milano Via C. Golgi 19 I-20133 Milan Italy

## Abstract

Bicyclo[6.1.0]nonyne carboxylic acid (BCN-COOH) is a valuable intermediate for the development of bioorthogonal click reagents. A convenient three-step synthesis of pure diastereomer *anti*-BCN-COOH is reported here, with an overall yield of 32% starting from 1,5-cyclooctadiene. To the best of our knowledge, this is the shortest route to *anti*-BCN-COOH known to date. The new method compares favourably with the optimised four-step synthesis based on previously reported data.

## Abstract 2

Synthetic availability of *anti*-BCN-COOH will benefit development of bioorthogonal click reagents.

## Graphical abstract

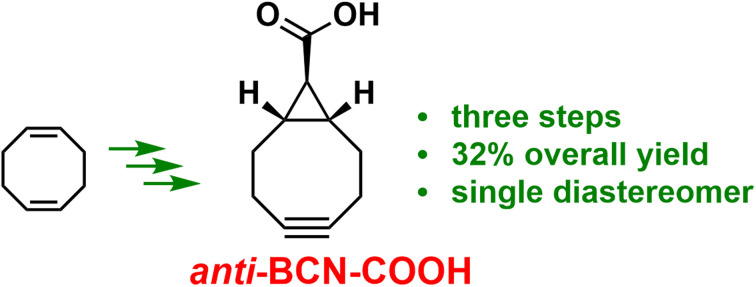

## Introduction

**Scheme 1:**
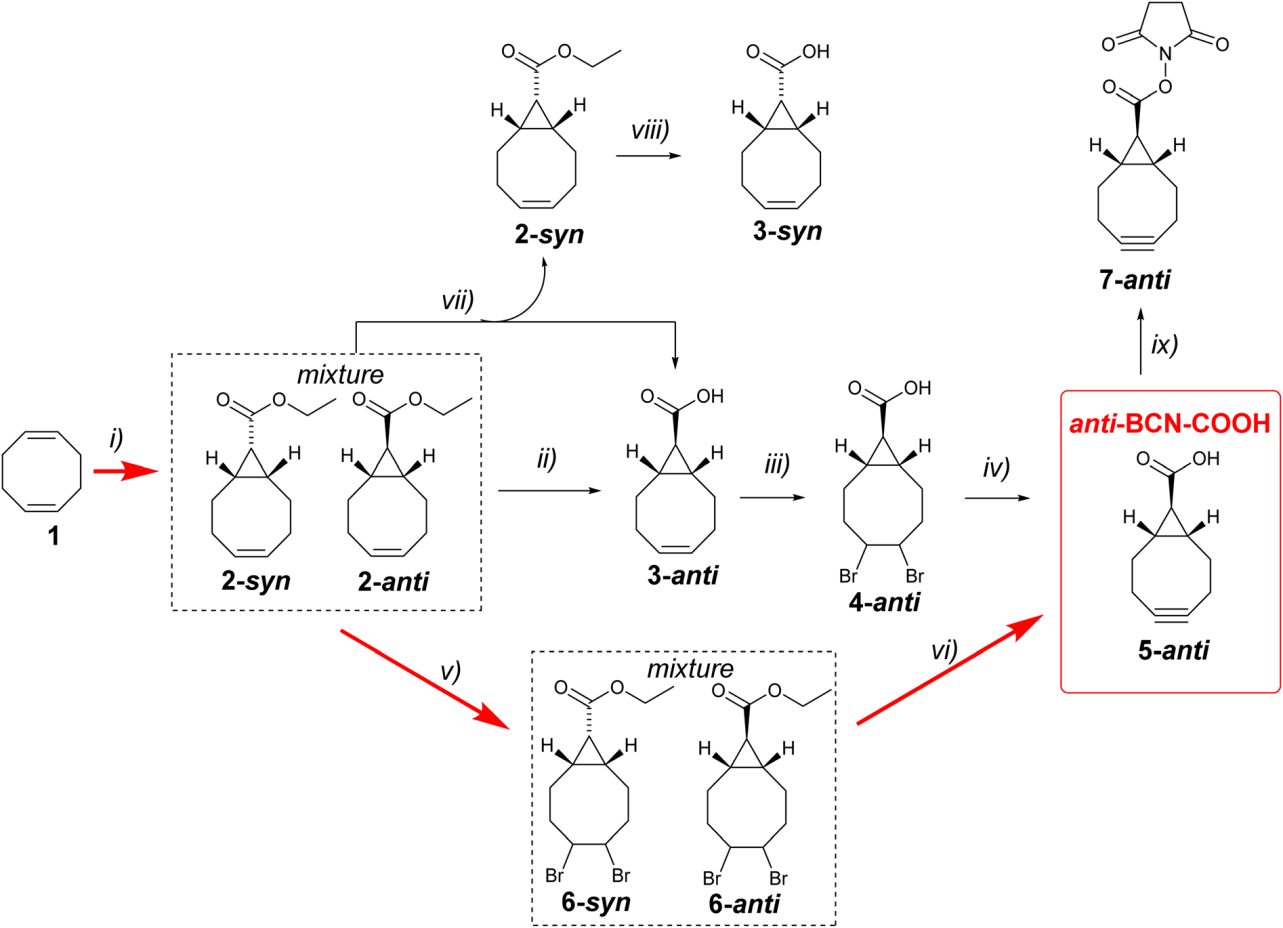
Reaction conditions: (i) ethyldiazoacetate, Rh_2_(OAc)_4_, 96%;^14^ (ii) THF (0.03% H_2_O), H_2_O, *t*-BuOK (1.5 eq.), RT, 17 h, 90%; (iii) Br_2_, chloroform, 0 °C, 73%; (iv) THF, *t*-BuOK (4.8 eq.), 40 °C 15 min, RT, 17 h, 44%; (v) Br_2_, chloroform, 0 °C, 89%; (vi) THF, *t*-BuOK (5 eq.), RT, 17 h, 37%; (vii) MeOH, H_2_O, LiOH (0.75 eq.), reflux, 30 min; (viii) MeOH, H_2_O, LiOH (1.5 eq.), reflux, 4 hours, 92%; (ix) *N*-hydroxysuccinimide, EDC·HCl, DCM, RT, 16 hours, 52%.

The success of bioorthogonal chemistry is underpinned by the development of click reagents. Strained alkyne bicyclo[6.1.0]non-4-yne (BCN) is extensively used in bioorthogonal reactions with azides (SPAAC),^1,2^ tetrazines (IEDDA),^3^ triazines (IEDDA),^4–6^ syndones,^7^ nitrones (SPANC),^8^ and tetrazoles.^9^ Due to the widespread application of BCN derivatives in bioorthogonal chemistry, developing the most efficient synthetic pathways to this valuable reagent is crucial. While hydroxymethyl-functionalized BCN^10^ is currently the most commonly used derivative in bioorthogonal chemistry, in 2021, bicyclo[6.1.0]nonyne carboxylic acid was reported by Wagner, Chaubet, and co-workers.^11^ The *syn*-isomer (*syn*-BCN-COOH) was prepared in five steps starting from 1,5-cyclooctadiene and was later successfully used in several click bioconjugations.^11,12^ The anti-isomer 5-*anti* (*anti*-BCN-COOH, Scheme 1) was previously mentioned as an intermediate in the synthesis of BCN linker derivatives for automated solid-phase oligonucleotide synthesis.^13^ However, the detailed experimental procedure for synthesising *anti*-BCN-COOH was not reported, and the compound was not fully characterised. In this short paper, we describe optimised synthesis based on known chemistry as well as a novel and efficient three-step synthesis of *anti*-BCN-COOH.

## Results and discussions

As a starting point, we decided to investigate the synthetic route previously mentioned by Karale *et al.*^13^ Cyclopropanation of 1,5-cyclooctadiene 1 using ethyl diazoacetate in the presence of a catalytic amount of Rh_2_(OAc)_4_ produces a mixture of *syn*- and *anti*-products 2-*syn* and 2-*anti* with approximately 45 : 55 selectivity (Scheme 1). Laborious chromatographic separation of the isomers is usually carried out at this stage. However, as elegantly shown by Fox and co-workers,^14^ ester hydrolysis under epimerising conditions leads exclusively to 3-*anti*, eliminating the need for chromatographic separation of isomers. We made minor modifications to the epimerisation procedure by conducting the reaction in chromatography-grade THF instead of dry diethyl ether and using only 1.5 equivalents of potassium *tert*-butoxide instead of three equivalents. We also carried out the reaction in air, without using inert or dry atmosphere. The optimised reaction worked well. The yield of compound 3-*anti* in 10 g scale reaction was comparable to the previously published yield (90%).

Next, 3-*anti* was brominated to give 4-*anti*, which was then used in the synthesis of the desired *anti*-BCN-COOH 5-*anti* by double dehydrobromination with potassium *tert*-butoxide. The product was characterised by ^1^H NMR, ^13^C NMR, and HRMS. Overall, this optimised four-step synthesis provides a good route to the desired *anti*-BCN-COOH. The final step is the most challenging due to the formation of unidentified byproducts with an Rf value similar to that of the desired 5-*anti*. Purification was achieved by first precipitating most of the byproducts in a 1 : 1 diethyl ether/hexane mixture, followed by purification using column chromatography.

After successfully completing the synthesis of the desired *anti*-BCN-COOH 5-*anti*, we aimed to further improve the process. Our hypothesis was that both the epimerisation and double dehydrobromination steps involve potassium *tert*-butoxide, so we decided to investigate whether these two steps could be combined into a single *in situ* procedure. The mixture of 2-*syn* and 2-*anti* was brominated to give a mixture of dibromo derivatives 6-*syn* and 6*-anti* in high yield. Subsequently, this mixture was reacted with five equivalents of potassium *tert*-butoxide in chromatography-grade THF (water content 0.03%) at room temperature for 14 hours in a flask exposed to air. In this way, epimerisation, hydrolysis and dehydrobromination are happening at the same time and gave after purification by column chromatography 5-*anti* in a 37% isolated yield. To the best of our knowledge, this is the shortest synthetic route to *anti*-BCN-COOH 5-*anti* reported to date.

The carboxylic acid may be activated for use in bioconjugation, as we exemplified here by the preparation of an NHS ester 7-*anti* (Scheme 1). It should be noted that alkenes 3-*syn* and 3-*anti* are valuable intermediates because they may be photochemically isomerised into their corresponding s-TCO derivatives,^15^ which are useful bioorthogonal reagents. While 3-*anti* can be easily obtained through epimerisation, the synthesis of 3-*syn* is more laborious and was initially based on chromatographic separation of an ester mixture 2-*syn* and 2-*anti*, followed by hydrolysis of each isomer. We observed that running the hydrolysis under identical conditions in a methanol–water mixture (3 : 1 v/v) in the presence of 1.5 molar equivalents of lithium hydroxide produced good yields of the corresponding acids, but the hydrolysis of ester 2-*syn* (4 hours) is significantly slower than that of 2-*anti* (30 minutes) presumably due to steric hindrances (ESI†).

Based on this observation, we developed a procedure to eliminate the laborious chromatographic separation of esters. By performing the hydrolysis of the ester mixture with 0.75 molar equivalents of lithium hydroxide in aqueous methanol for 30 minutes, we obtained a mixture of the water-soluble lithium salt of acid 3-*anti* and ester 2-*syn*, which is soluble in diethyl ether. Simple extraction using diethyl ether and water yielded pure 2-*syn* and 95% pure 3-*anti*. The ester 2-*syn* was then converted into 3-*syn* by more persistent hydrolysis (Scheme 1). It should be noted that bromination of 2-*syn* followed by treatment with potassium *tert*-butoxide in wet THF yielded not 5-*syn* but 5-*anti* as expected.

## Conclusion

A novel three-step synthesis of the strained alkyne *anti*-BCN-COOH 5-*anti* is reported. This concise route does not require the use of dry solvents or chromatographic separation of diastereoisomers. We hope that the synthetic accessibility of this important click reagent will benefit the further development of bioorthogonal chemistry.

## Experimental

### General

All solvents and reagents were purchased from commercial suppliers and used without further purification. NMR spectra were recorded on a JEOL ECS400FT Delta spectrometer. Chemical shifts are reported in parts per million (ppm) relative either to a tetramethylsilane internal standard or to a residual solvent peak. HRMS analysis was performed on LTQ Orbitrap XL Thermo Scientific. The mixture esters 2-*syn* and 2-*anti* was prepared as described previously^14^ (Reaction i).

### Reaction ii. Synthesis of acid 3-*anti* by ester hydrolysis under epimerising conditions^14^

A mixture of the esters 2-*syn* and 2-*anti* (10.8 g, 56 mmol) was dissolved in THF (50 mL, Fisher Scientific, chromatography GPC grade, 0.03% water content). 1 M solution of potassium *tert*-butoxide in THF (85 mL, 85 mmol) was added. The mixture was stirred at room temperature for 30 min. Water (750 μL, 42 mmol, 0.75 eq.) was added in five equal (150 μL) portions waiting for 10 minutes between each addition. The mixture was then stirred for 17 hours (the flask was open to air). Water (100 mL) and hexane (100 mL) were added, and the mixture was separated. The aqueous layer was rotary evaporated to a volume of 30 mL. 2 M aqueous HCl (45 mL, 90 mmol) was added causing formation of a solid. The solid was filtered off, washed with water, and dried by running the suction for 2 hours then keeping the solid on a watch glass in the fume hood overnight. Yield 8.4 g (90%). NMR data is identical to previously published.^14^

### Reaction iii. Synthesis of dibromo derivative 4-*anti* (ref. 13)

A solution of acid 3-*anti* (2.50 g, 15 mmol, 1 eq.) in chloroform (25 mL) was cooled to 0 °C using an ice/water bath. A solution of elemental bromine (810 μL, 15.8 mmol, 1 eq.) in chloroform (25 mL) was added dropwise to the reaction mixture over period of 30 min using a pressure equalised addition funnel. The reaction stirred for 15 min. 1 M sodium thiosulphate (15 mL) and chloroform (20 mL) were added and the mixture was separated. The aqueous phase was washed with chloroform (25 mL). The combined organic phase was washed with brine (50 mL), dried over MgSO_4_, filtered and evaporated to dryness to give dibromo derivative 4-*anti* as a colourless solid (3.57 g, 73%). ^1^H NMR (400 MHz, CDCl3) *δ* 4.84–4.78 (m, 2H, 2 × BrC-H), 2.75–2.62 (m, 2H), 2.34–2.25 (m, 1H), 2.13–2.08 (m, 3H), 1.80–1.72 (m, 2H), 1.82–1.69 (m, 2H), 1.54–1.38 (m, 2H), 1.22–1.20 (m, 1H). ^13^C NMR (100 MHz, CDCl_3_) *δ* 197.7, 55.5, 52.1, 34.9, 34.3, 29.2, 27.5, 26.9, 23.9, 23.0.

### Reaction iv. Synthesis of *anti*-bicyclo[6.1.0]nonyne carboxylic acid 5-*anti* from dibromo derivative 4-*anti*

The dibromo derivative 4-*anti* (5.4 g, 16.5 mmol, 1 eq.) was dissolved in dry THF (30 mL). The solution was transferred into a dropping funnel and added dropwise to a cold (−40 °C, acetonitrile/dry ice bath) 1 M solution of potassium *tert*-butoxide in THF (80 mL, 80 mmol, 4.8 eq.). The mixture was stirred at −40 °C for 15 minutes, then the bath was removed. The mixture was stirred at RT for 17 hours. 1 M solution of KHSO_4_ (50 mL, 50 mmol) was added until acidic pH. Brine (50 mL) and diethyl ether (50 mL) were added. The mixture was transferred into a separating funnel and separated. The organic layer was washed with brine (50 mL), dried over MgSO_4_, gravity filtered and evaporated. The solid was dissolved in diethyl ether (20 mL). Hexane (20 mL) was added, and mixture was left kept at RT for 1 hour (biproduct precipitated). The mixture was filtered. The filtrate was applied to a silica gel column equilibrated in a mixture of diethyl ether/hexane, 1/1, v/v. The column was eluted with diethyl ether/hexane, 1/1, v/v (Rf value of the product 0.18). The fractions containing pure product were combined and evaporated to dryness to give the desired product. Yield 1.2 g (44%) ^1^H NMR (400 MHz, DMSO-d_6_) *δ* 11.97 (s, 1H), 2.31–2.14 (m, 4H), 2.10–2.01 (m, 2H), 1.40–1.29 (m, 2H), 1.26–1.18 (m, 2H), 1.08 (t, *J* = 5.2 Hz, 1H). ^13^C NMR (100 MHz, DMSO-d_6_) *δ* 175.6, 99.3, 32.5, 27.3, 26.1, 20.9 HRMS (ESI): *m*/*z* calcd for [M + H] 165.09155, found 165.09076.

### Reaction v. Synthesis of a mixture of dibromo derivatives 6-*syn* and 6-*anti*

A mixture of esters 2-*syn* and 2-*anti* (6 g, 30.9 mmol, 1.0 eq.) in chloroform (60 mL) was cooled to 0 °C and stirred for 5 min. A pressure equalised addition funnel containing a solution of elemental bromine (5 g, 31.3 mmol, 1.01 eq.) in chloroform (30 mL). The bromine solution was added dropwise to the reaction mixture over 10 min. The reaction stirred for an additional 5 min and then mixed with 1 M aqueous sodium thiosulphate (30 mL). The organic layer was separated and washed with brine (30 mL), dried over MgSO_4_, gravity filtered and evaporated to dryness to give a mixture of dibromo derivatives 6-*syn* and 6-*anti* as a colourless solid. Yield 9.7 g (89%). The product was used in the next step without any further purification. The copy of NMR of this mixture is available in supplementary information (Fig. S9†).

### Reaction vi. Synthesis of acid 5-*anti* from the mixture of dibromo derivatives 6-*syn* and 6-*anti*

A solution of the mixture of dibromo derivatives 6-*syn* and 6-*anti* (3.54 g, 10 mmol, 1.0 eq.) in THF (30 mL, Fisher Scientific, chromatography GPC grade, 0.03% water, 9 mg, 0.5 mmol) was cooled to 0 °C. 1 M solution of potassium *tert*-butoxide in THF (50 mL, 50 mmol) was added dropwise. After the addition is complete, the ice bath was removed, and the mixture was stirred at room temperature for 17 hours. Water (50 mL) was added and the mixture was stirred for 30 minutes. Hexane (50 mL) was added, and the mixture was separated. The aqueous phase was washed with diethyl ether (50 mL) and then acidified with 1 M KHSO_4_ (40 mL). Diethyl ether (50 mL) was added and the mixture was separated. The aqueous phase was washed with diethyl ether (50 mL). The combined organic phase was dried over MgSO_4_, gravity filtered and evaporated to dryness. The product was purified by column chromatography (NOTE 1) on silica gel using a diethyl ether/hexane 1/1 mixture as an eluent (Rf value of the product). Yield 630 mg (37%). Characterisation data is identical to the described above.

### Reaction vii. Synthesis of acid 3-*anti* and ester 2-*syn* by selective hydrolysis of 2-*syn*/2-*anti* mixture

To a solution of the mixture of esters 2-*syn*/2-*anti* (11.7 g, 60 mmol, 1.0 eq.) in methanol (60 mL), a warm (50 °C) solution of lithium hydroxide monohydrate (1.9 g, 45.0 mmol, 0.75 eq.) in water (20 mL) was added. The reaction mixture was stirred and heated under reflux for 30 minutes (TLCs carried out to check progression of reaction). The mixture was evaporated to a volume of approximately 15 mL. The residue was dissolved in water (70 mL). The solution was extracted with diethyl ether (3 × 60 mL). The organic layer was dried with magnesium sulfate, gravity filtered and evaporated to give ester 2-*syn*. Yield 3.35 g (29%). NMR data is identical to previously published (Fig. S1†).^10^

The aqueous layer was placed in a round-bottomed flask and acidified by adding 2 M HCl (25 mL, 46 mmol), causing formation of the semisolid. The flask was attached to rotary evaporator and heated at 50 °C in vacuum for 5 minutes. The mixture was allowed to cool to RT. The solid was filtered off, washed with water to give acid 3-*anti* as a colourless solid. Yield 6.3 g (63%, 95% pure, 5% of 3-*syn*). ^1^H NMR (400 MHz, CDCl_3_) *δ* 5.66–5.58 (m, 2H), 2.29–2.26 (m, 2H), 2.21–2.17 (m, 2H), 2.09–2.04 (m, 2H), 1.63–1.58 (m, 2H), 1.50–1.47 (m, 2H), 1.19–1.18 (m, 1H). ^13^C NMR (100 MHz, CDCl_3_) *δ* 180.8, 130.0, 31.1, 28.9, 27.7, 26.7.

### Reaction viii. Synthesis of acid 3-*syn* by hydrolysis of ester 2-*syn*

To a solution of the ester 2-*syn* (4.91 g, 25.3 mmol, 1.0 eq.) in methanol (60 mL), a warm solution of lithium hydroxide monohydrate (1.6 g, 38.0 mmol, 1.5 eq.) in water (30 mL) were added. The reaction mixture was stirred and heated under reflux for 4 hours. The mixture was rotary evaporated to a volume of approximately 10 mL and diluted with water (150 mL). After that 2 M HCl (20 mL, 40 mmol) was added to the mixture, causing formation of the solid. The solid was filtered off, washed with water to give acid 3-*syn* acid as a colourless solid. Yield 3.86 (92%). ^1^H NMR (400 MHz, CDCl_3_) *δ* 5.64–5.56 (m, 2H), 2.50–2.45 (m, 2H), 2.24–2.16 (m, 2H), 2.10–2.00 (m, 2H), 1.72 (t, *J* = 8.0, 1H), 1.52–1.43 (m, 2H); ^13^C NMR (100 MHz, CDCl_3_) *δ* 178.1, 129.6, 27.1, 25.4, 22.7, 21.0.

### Reaction ix. Synthesis of NHS-ester 7*-anti*

The carboxylic acid *anti*-BCN-COOH 5*-anti* (900 mg, 5.49 mmol) was dissolved in DCM (30 mL). *N*-Hydroxysuccinimide (950 mg, 8.24, 1.5 eq.) and EDC hydrochloride (1.58 g, 8.24 mmol, 1.5 eq.) and were sequentially added. The clear solution was stirred at RT overnight. Water (30 mL) was added, and the mixture was separated. The organic phase was dried over MgSO_4_, gravity filtered and evaporated to dryness to give the crude product. The product was then purified by column chromatography on silica gel using initially DCM then DCM/diethyl ether/hexane, 1/1/1, v/v/v mixture as an eluent. Yield 740 mg (52%). ^1^H NMR (400 MHz, CDCl_3_) *δ* 2.82 (s, 4H), 2.55–2.51 (m, 2H), 2.38–2.31 (m, 2H), 2.23–2.19 (m, 2H), 1.70–1.67 (m, 2H), 1.50–1.43 (m, 3H). ^13^C NMR (100 MHz, CDCl_3_) *δ* 169.4, 169.3, 98.4, 32.3, 29.7, 25.6, 22.9, 20.7. HRMS (ESI): *m*/*z* calcd for [M + H] 262.10793, found 262.10730.

## Data availability

The data supporting this article have been included as part of the ESI.†

## Conflicts of interest

There are no conflicts to declare.

## Supplementary Material

RA-014-D4RA06708H-s001
