# Supplementary material for: A concise synthesis of anti-bicyclo[6.1.0]nonyne carboxylic acid
Source: RSC Adv. 2024 Nov 21;14(50):37400–3. doi: 10.1039/d4ra06708h (PMC11580154; doi:10.1039/d4ra06708h)
Supplement: RA-014-D4RA06708H-s001 [file RA-014-D4RA06708H-s001.pdf]

Supplementary Information (SI) for RSC Advances.
This journal is © The Royal Society of Chemistry 2024

Supplementary information

A concise synthesis of *anti*-bicyclo[6.1.0]nonyne carboxylic acid.

Mackenzie Weir,[a] Beatrice Vaccari,[a,b] Aidan Matthews,[a] Graeme Turnbull[a] and Valery Kozhevnikov*[a]

[a] Department of Applied Sciences, Northumbria University, Ellison Building, Newcastle upon Tyne, NE1 8ST, U.K.

[b] Dipartimento di Chimica dell'Università degli Studi di Milano, Via C. Golgi 19, I-20133, Milan, Italy

Synthesis of acid 3-*anti* by ester hydrolysis of ester 2-*anti*

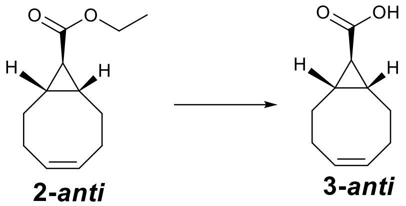

The ester **2-*anti*** (4.91 g, 25.3 mmol, 1 eq.) was dissolved in ethanol (60 ml). A warm (50°C) solution of lithium hydroxide monohydrate (1.6 g, 38.0 mmol, 1.5 eq.) in water (30 ml) was added. The reaction mixture was stirred and heated under reflux for **30 minutes**. The mixture was allowed to cool to RT and evaporated. The residue was diluted with water to a volume of 150 mL. 2M aqueous HCl (20 mL, 40 mmol) was added, causing formation of the solid. The solid was filtered off, washed with water to give acid **3-*anti*** as a colourless solid. Yield 3.5 g (84%). NMR data is identical to previously published (Figure S4).[1]

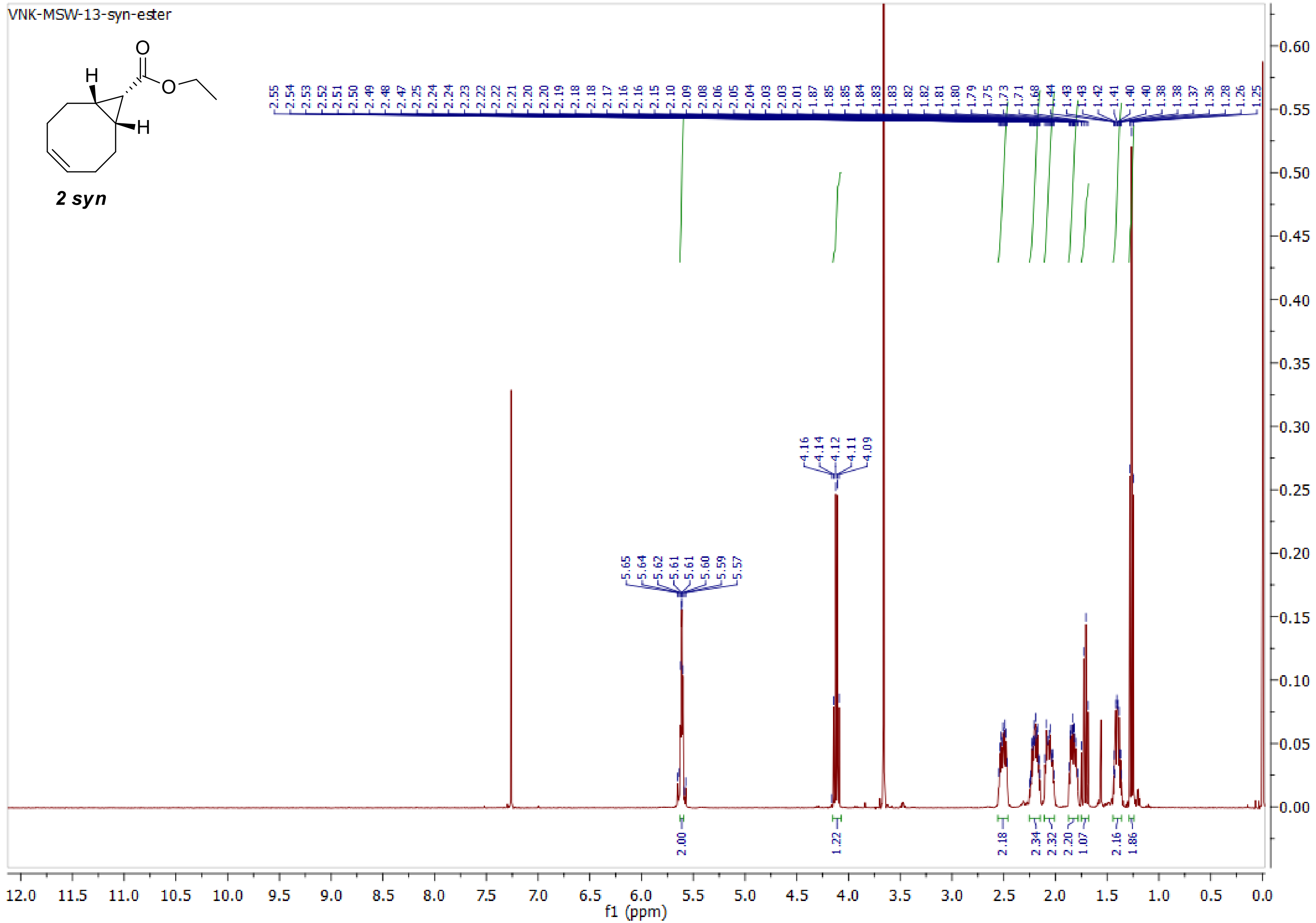

Figure S1. ^{1}H NMR spectrum of ***2-syn*** in $CDCl_3$.(1) O'Brien, J. G. K.; Chintala, S. R.; Fox, J. M. Stereoselective Synthesis of Bicyclo[6.1.0]nonene Precursors of the Bioorthogonal Reagents s-TCO and BCN. *The Journal of Organic Chemistry* **2018**, *83* (14), 7500-7503. DOI: 10.1021/acs.joc.7b02329.

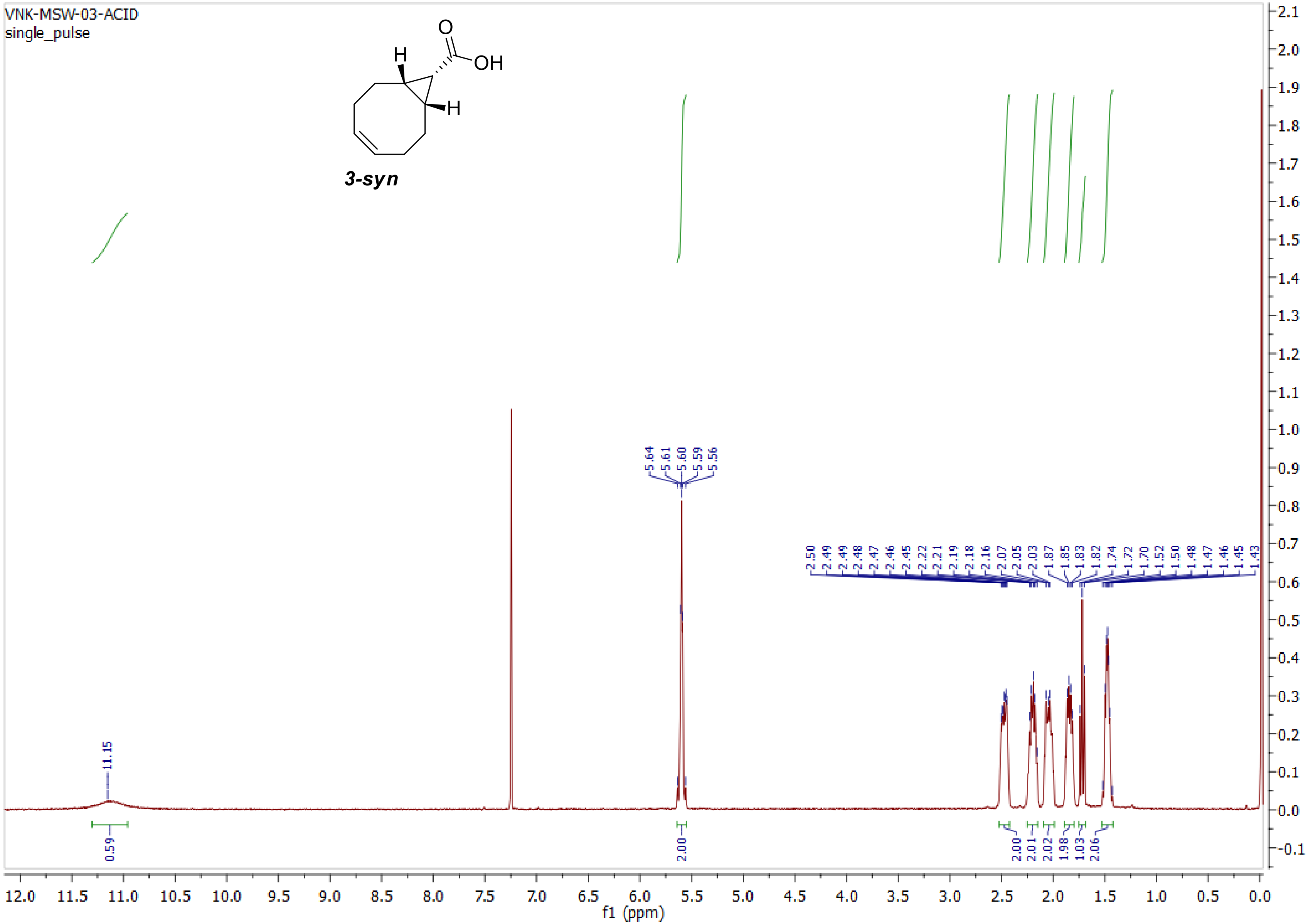

Figure S2: ^{1}H NMR spectrum of ***3-syn*** in $CDCl_3$.

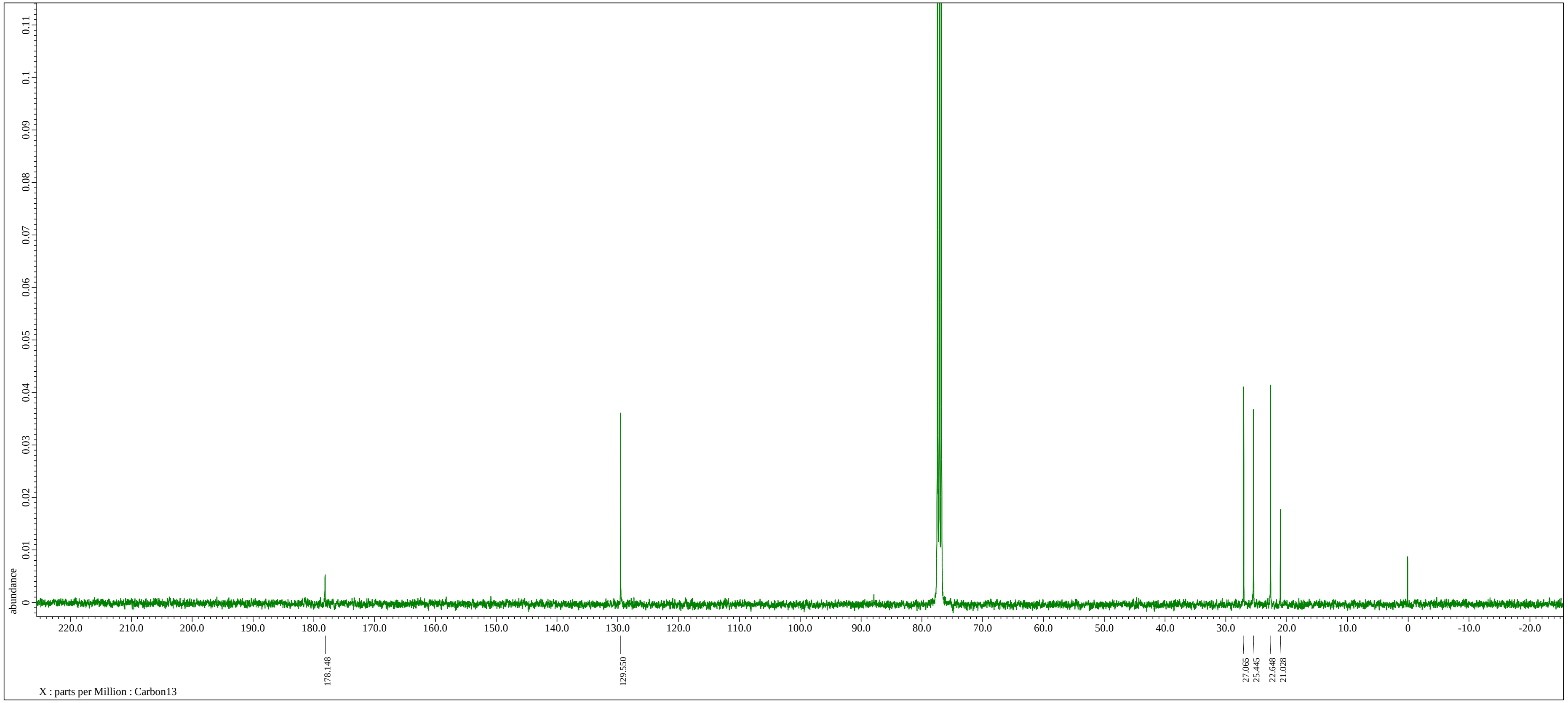

Figure S3: ^{13}C NMR spectrum of ***3-syn*** in $CDCl_3$.

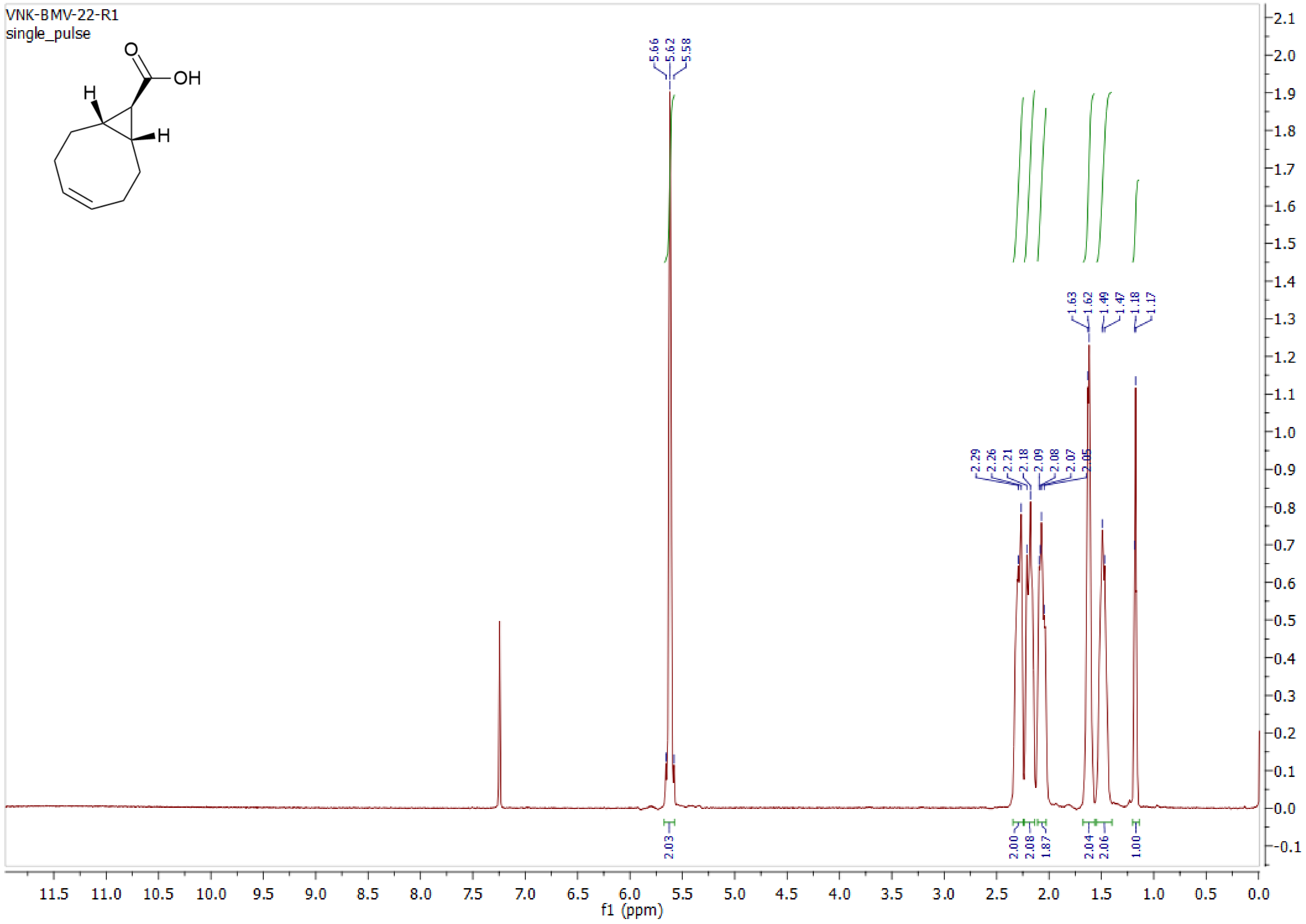

Figure S4: ^{1}H NMR spectrum of **3-*anti*** in $CDCl_3$.

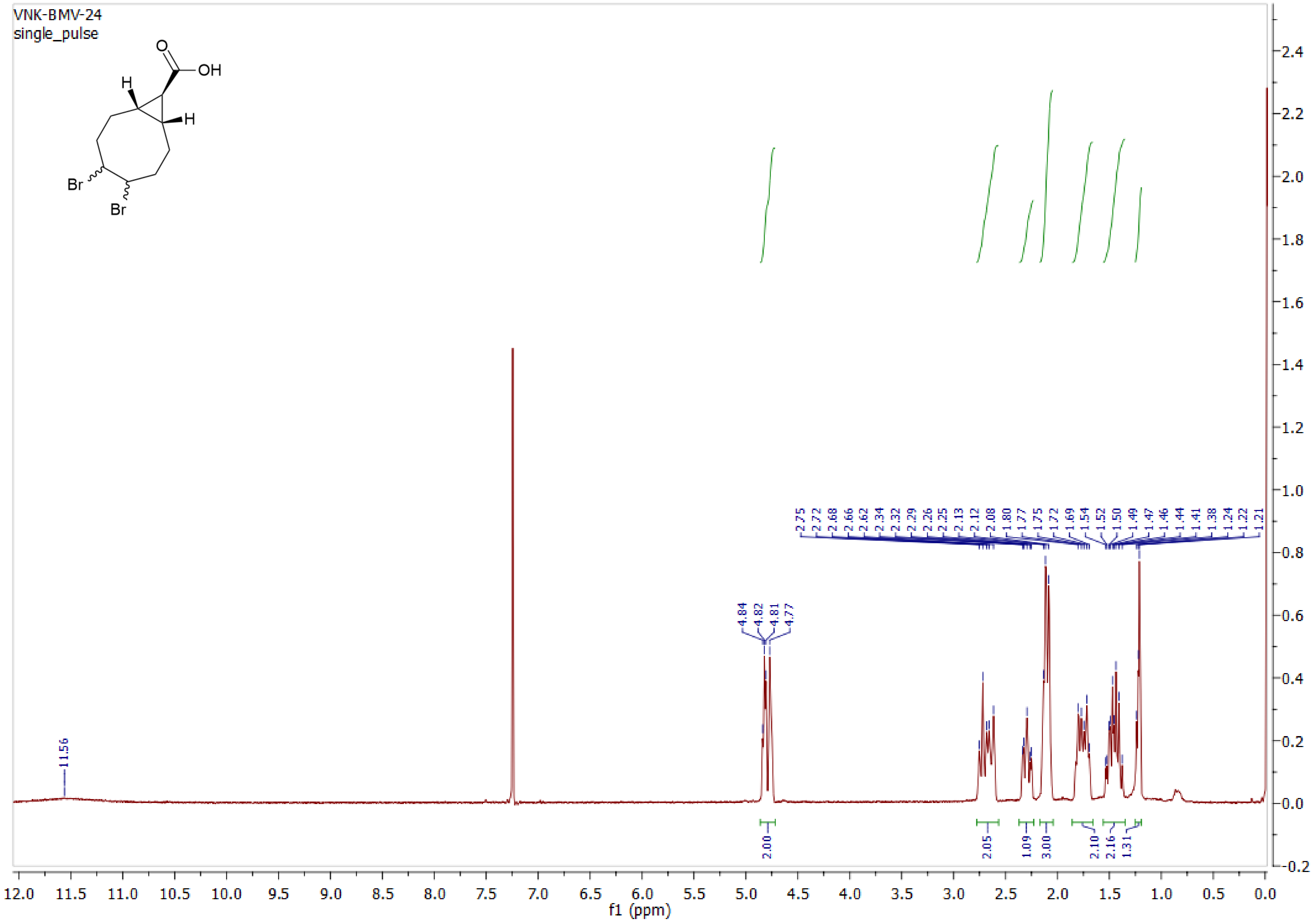

Figure S5: ^{1}H NMR spectrum of ***4-anti*** in $CDCl_3$.

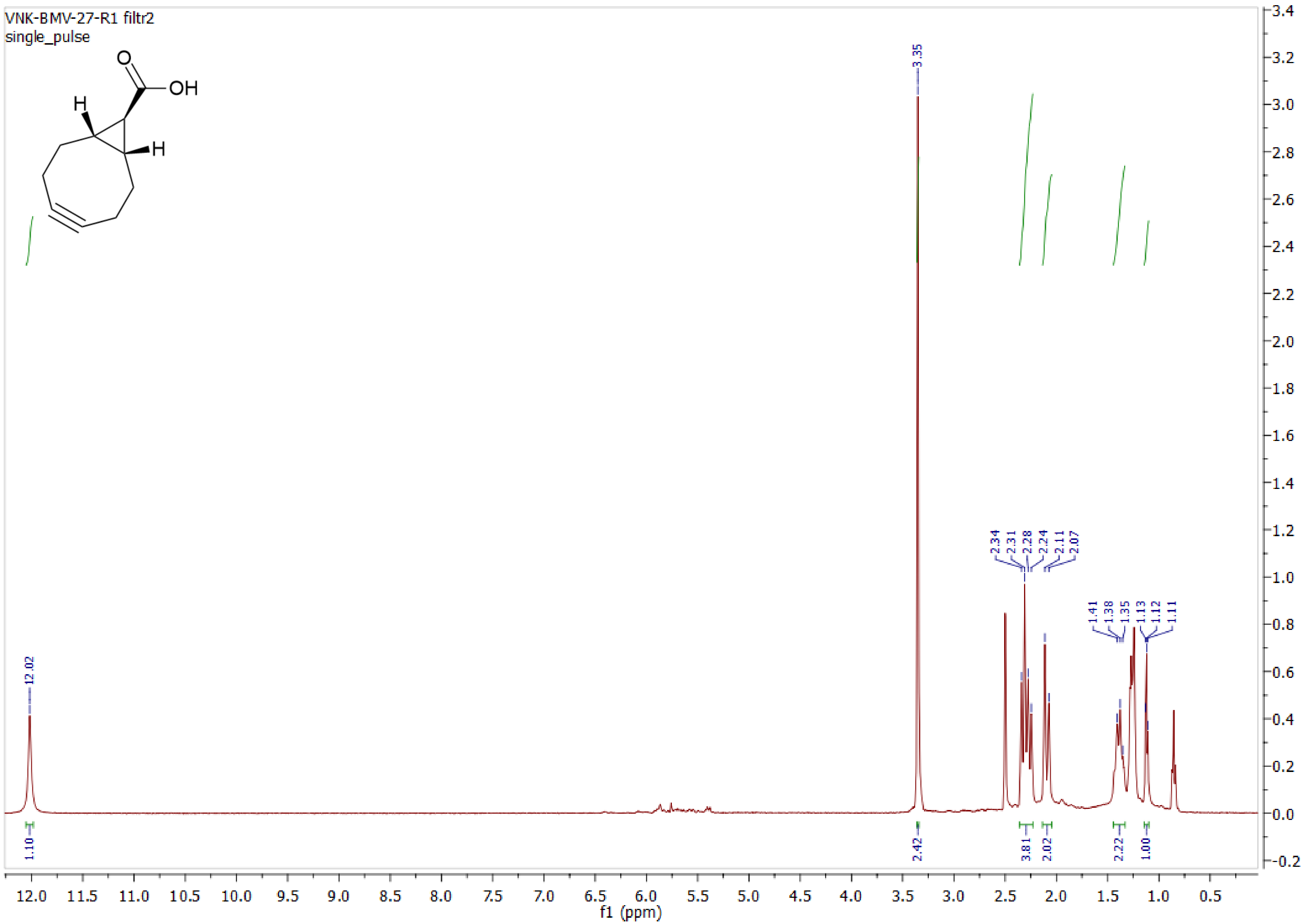

Figure S6: ^{1}H NMR spectrum of **5-*anti*** in DMSO-D_6.

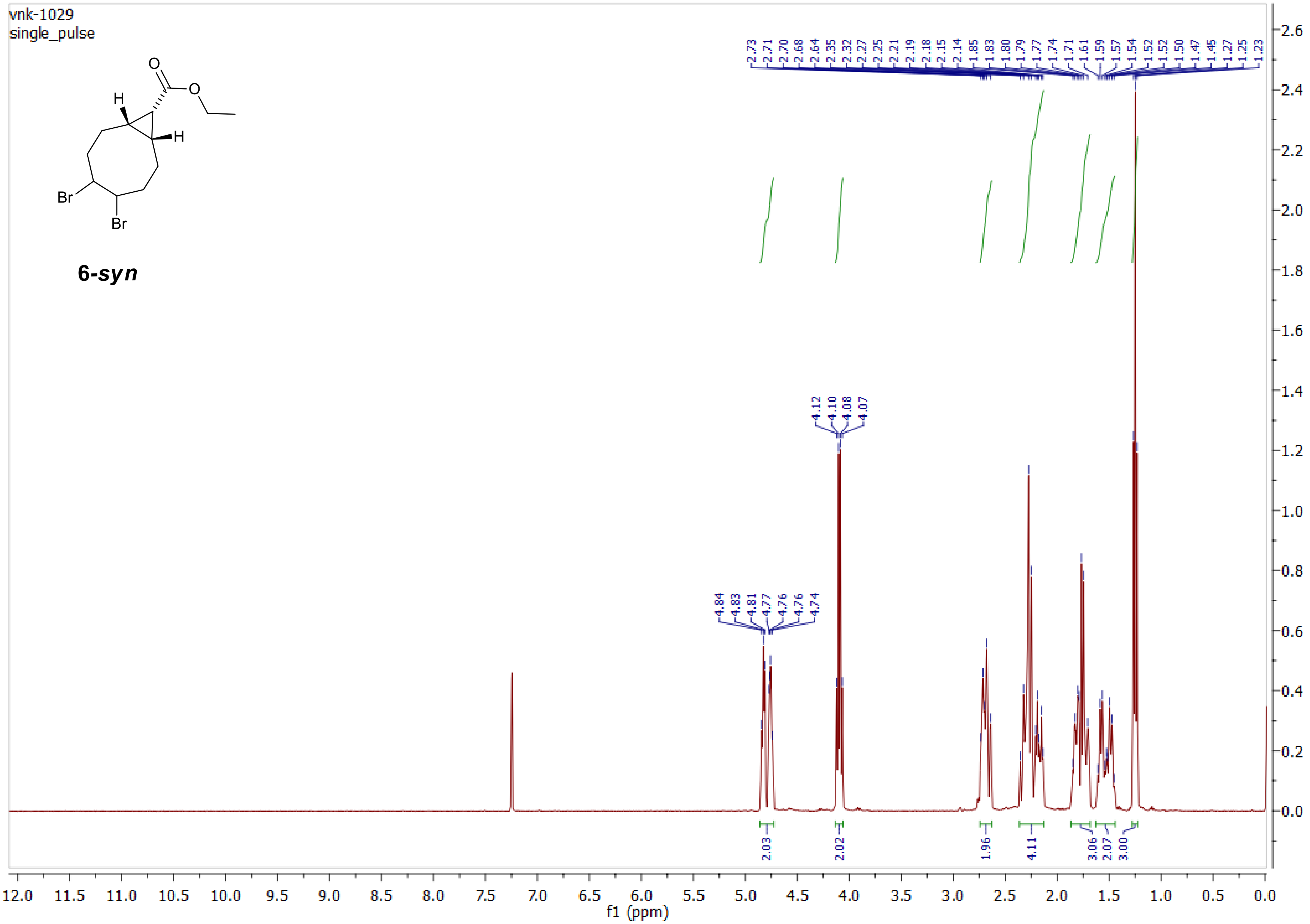

Figure S7: ^{1}H NMR spectrum of ***6-syn*** in $CDCl_3$.

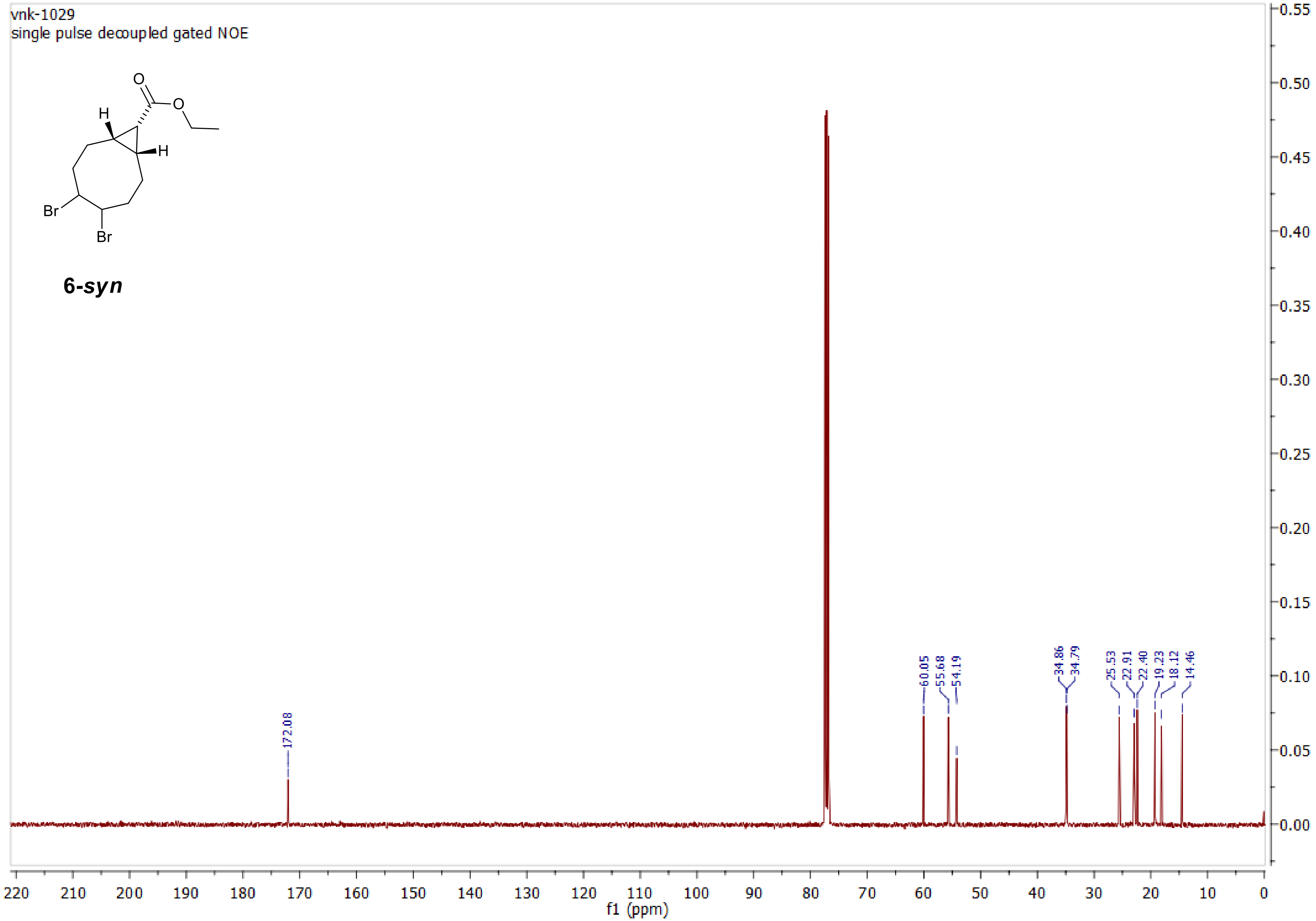

Figure S8: ^{13}C NMR spectrum of ***6-syn*** in $CDCl_3$.

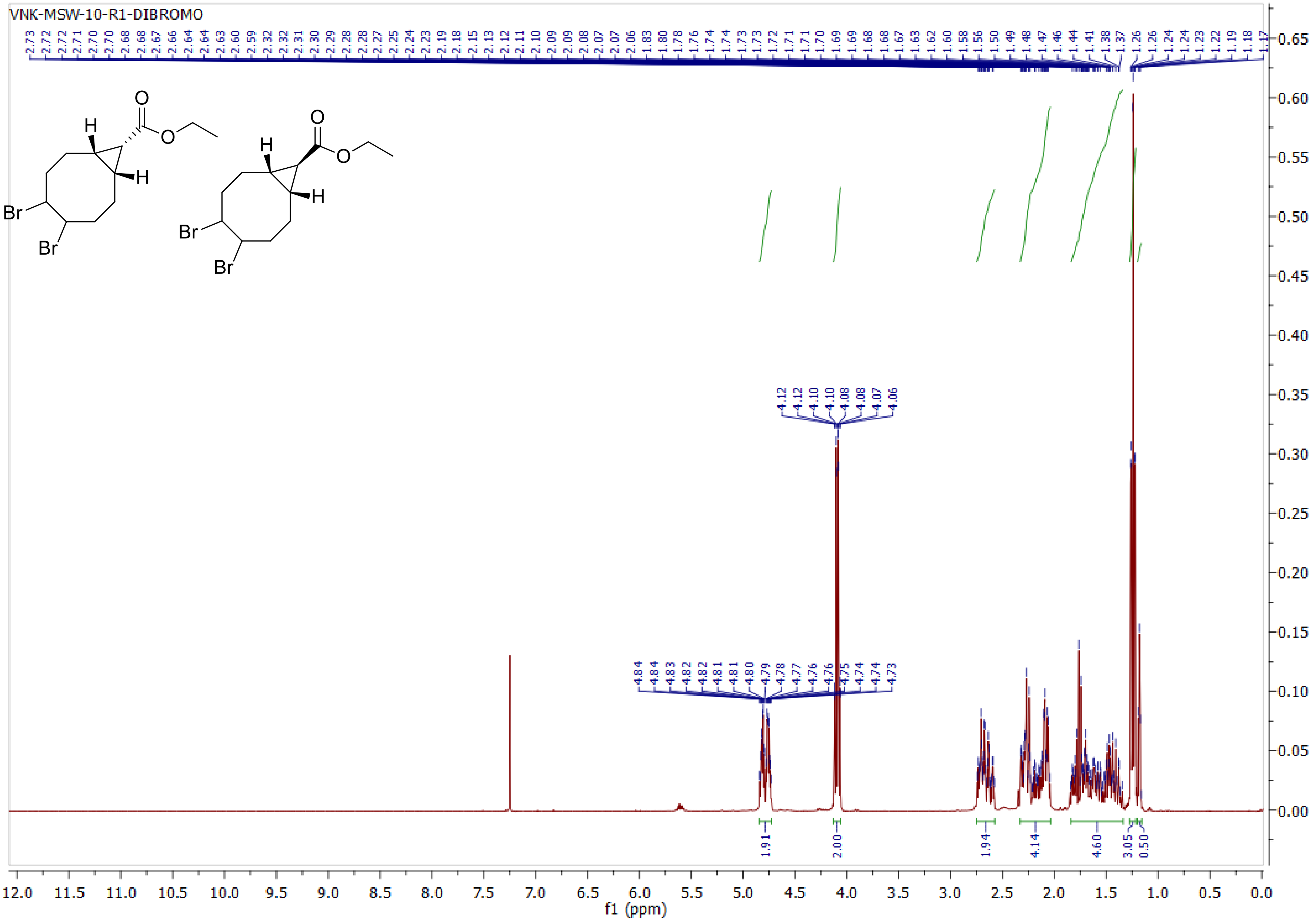

Figure S9: ^{1}H NMR spectrum of ***VNK-MSW-10-R1 (6-syn 6-anti)*** in $CDCl_3$.

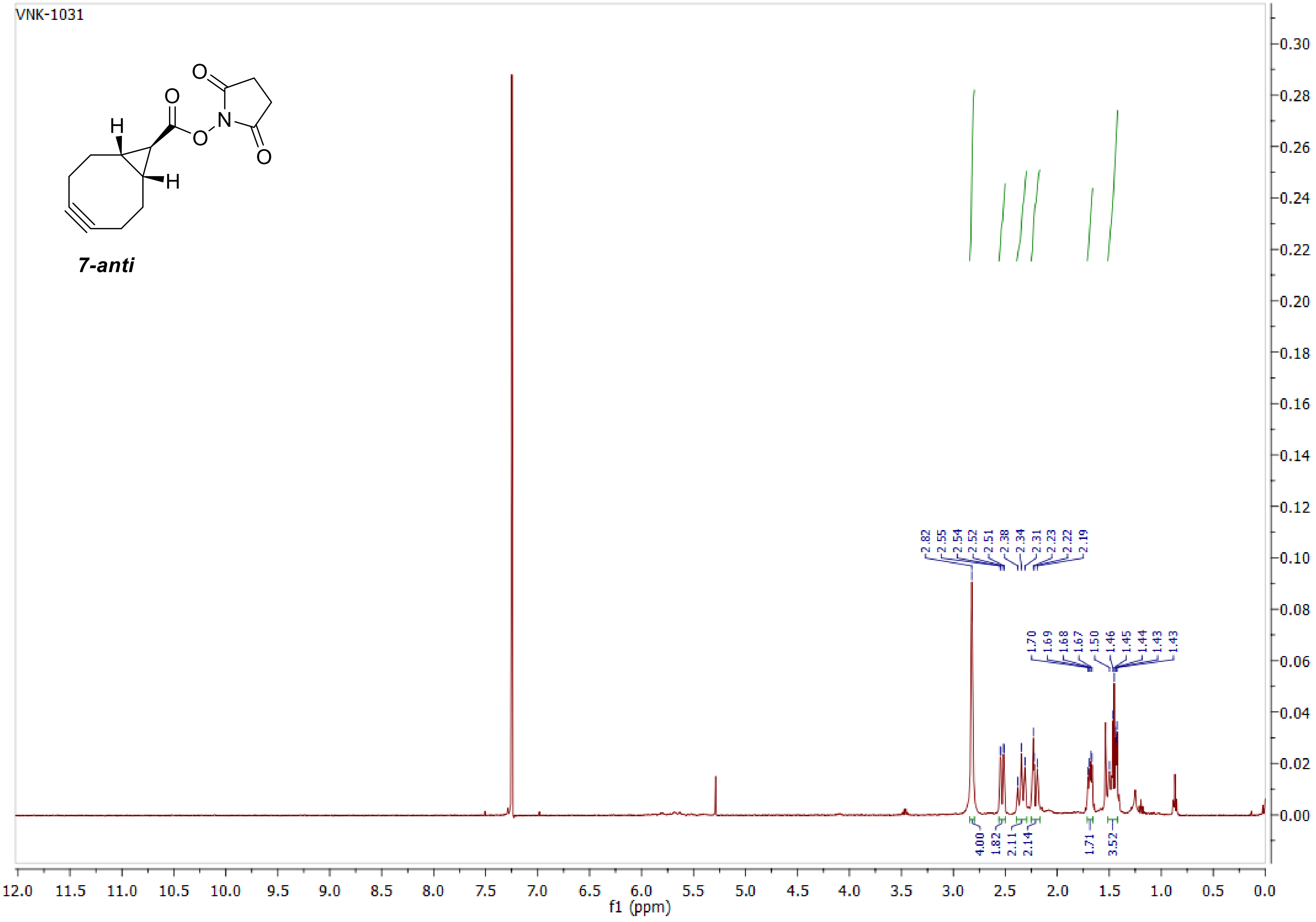

Figure S10: ^{1}H NMR spectrum of 7-***anti*** in $CDCl_3$.

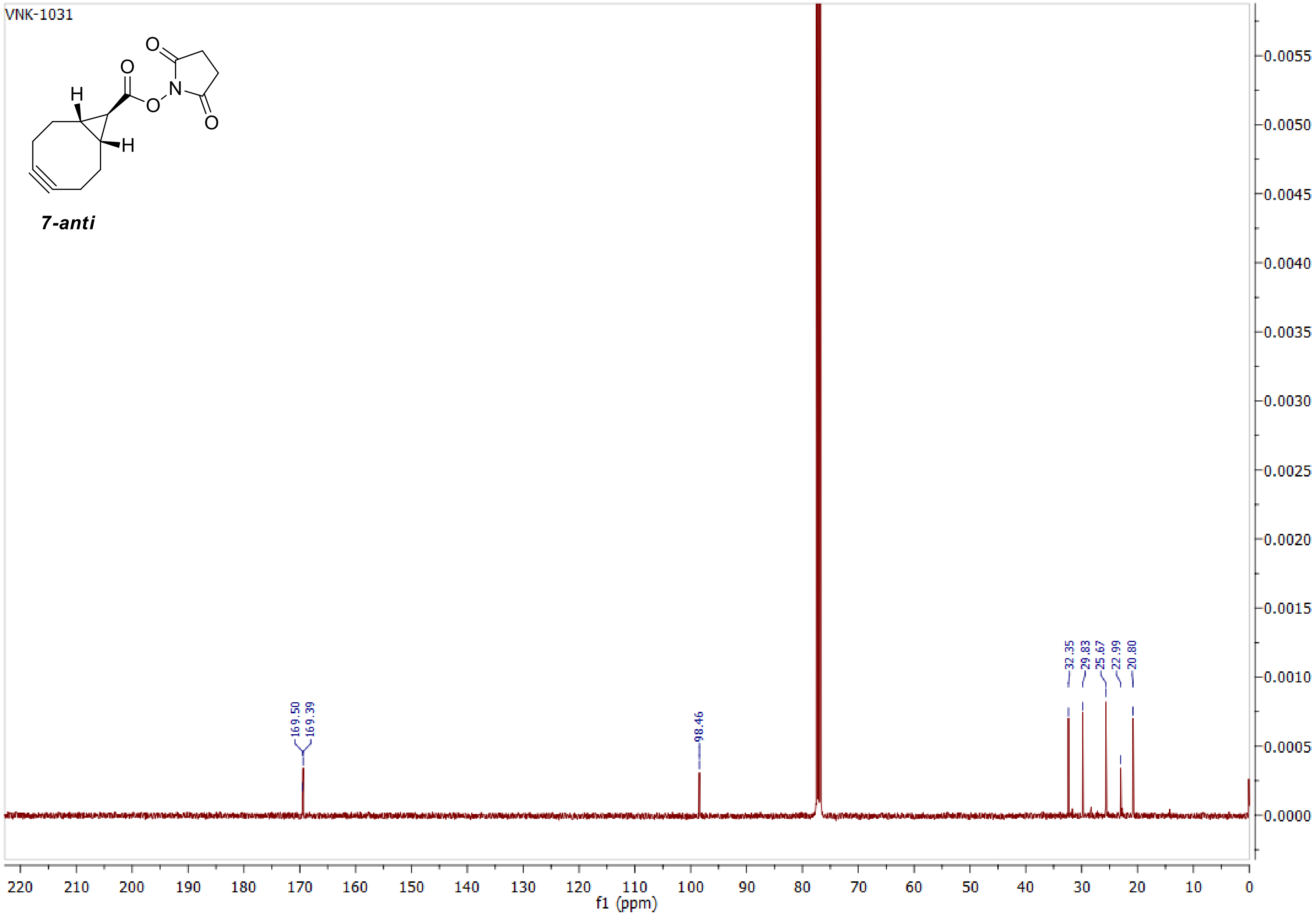

Figure S11: ^{13}C NMR spectrum of ***7-anti*** in $CDCl_3$.
